# Polycyclic Aromatic Hydrocarbons (PAHs) and Phthalate Esters (PAEs) in the Farmed Fishes from Khanh Hoa, Viet Nam: Level and Health Risk Assessment

**DOI:** 10.3390/foods14203518

**Published:** 2025-10-16

**Authors:** Xuan-Vy Nguyen, Trung-Du Hoang, Nhu-Thuy Nguyen-Nhat, Quoc-Hoi Nguyen, Xuan-Thuy Nguyen, Trung-Hieu Nguyen, Si Hai Trinh Truong, My-Ngan T. Nguyen, Viet-Ha Dao

**Affiliations:** 1 Institution of Oceanography, Vietnam Academy of Science and Technology, 01 Cau Da, Nha Trang Ward 650000, Khanh Hoa, Vietnam; hoangtrungdu1@io.vast.vn (T.-D.H.); nhat.thuy.174@gmail.com (N.-T.N.-N.); xuanthuynguyen1293@gmail.com (X.-T.N.); trunghieuhdh@gmail.com (T.-H.N.); haitrinh@io.vast.vn (S.H.T.T.); myngan.ion@gmail.com (M.-N.T.N.); daovietha69@gmail.com (V.-H.D.); 2 Faculty of Biology, Graduate University of Science and Technology, Vietnam Academy of Science and Technology, 18 Hoang Quoc Việt, Cau Giay, Ha Noi 65000, Vietnam; quochoi1804@gmail.com

**Keywords:** health risk, farmed fishes, Khanh Hoa, PAEs, phthalates

## Abstract

Phthalic acid esters (PAEs) and polycyclic aromatic hydrocarbons (PAHs) are known to potentially impact both marine organisms and human health through the consumption of fish and seafood. In this study, the concentrations of 12 priority PAHs and 6 PAEs were analyzed in the tissues of 76 samples of five farmed fish species, including *Litopenaeus vannamei* (crustacean), *Babylonia areolata*, *Marcia hiantina* (mollusk), *Trachinotus blochii*, and *Epinephelus lanceolatus* (fish), collected from four coastal sites in Khanh Hoa province. Freeze-dried tissue was extracted using water bath ultrasonication with an acetone/n-hexane mixture. A triple quadrupole gas chromatograph–mass spectrometer (GC-MS/MS) was used for the analyses. The results showed that the total PAHs had low contamination levels. Among the PAEs, bis(2-ethylhexyl) phthalate (DEHP) exhibited the highest concentrations. The calculated hazard index (HI) for PAEs suggested no significant health risk. Six PAHs were detected, ranging from 9.14 µg kg^−1^ in Pacific white shrimp to 47.34 µg kg^−1^ in cockle. The incremental lifetime cancer risk (ILCR) values for PAHs in some samples exceeded the acceptable safety threshold. In the future, natural fish, environmental samples (seawater and marine sediment), and other information on natural conditions will be collected for analyses. This is the first report on the levels and health risks of PAEs and PAHs in farmed fishes along the Khanh Hoa coast.

## 1. Introduction

Polycyclic aromatic hydrocarbons (PAHs) are lipophilic molecules, consisting of two or more fused, stable aromatic rings of carbon and hydrogen [1]. Polycyclic aromatic hydrocarbons (PAHs) are lipophilic molecules, consisting of two or more fused, stable aromatic rings of carbon and hydrogen [1]. PAHs are products of combustion, agricultural production, and transformation processes; they are derived from fossil fuels and synthesized by some organisms. They can be naturally found in the environment [2]. PAH pollutants are characterized as highly toxic, teratogenic, mutagenic, immunotoxicogenic, and carcinogenic to any life forms [3]. According to Duran and Cravo-Laureau [2], the entry of PAHs into the marine environment happens through chronic pollution and acute pollution. In recent decades, plastic pollution emerged as a major environmental issue. Worldwide, plastic production increased dramatically from 1.5 million tons in 1950 to 390.7 million tons by 2021 [4]. Alfaro-Núñez et al. [5] estimated that at least 5.25 trillion plastic particles have been disposed into the oceans. Plastic particles are highly persistent in the marine environment, and they are accumulating with an increasing rate [6,7]. Several studies revealed that marine microplastics serve as vectors of ocean pollutants and pose significant risks to marine organisms as well as human health [8,9]. Tang et al. [10] showed that there is a strong correlation between plastic films and various types of PAHs, including 3- and 4-ring PAHs in the coastal sediment.

In the manufacturing and processing of plastic products, phthalate esters (PAEs) are widely used. Previous studies revealed that PAEs are endocrine-disrupting chemicals that showed potential health risks to animals and humans [11,12]. Nowadays, thirty types of PAEs are identified, and six of them are priority pollutants [13]. According to Grmasha et al. [14], the marine sediments are reservoirs for the migration and transformation of both PAHs and PAEs. Wherever there is a high accumulation of plastic debris, concentrations of di(2-ethylhexyl) phthalate (DEHP) in the bottom layers of the water column show a high concentration [15]. PAHs and PAEs are easily absorbed by various marine organisms [16,17]. More than 50% of PAHs are potentially carcinogenic to humans [18]. Therefore, seafood should be monitored for PAH concentration due to its potential health risks via consumption [19]. For phthalate esters (PAEs), the International Agency for Research on Cancer (IARC) suggested that butyl benzyl phthalate (BBP) and DEHP are possibly carcinogenic to humans. In addition, dimethyl phthalate (DMP), diethyl phthalate (DEP), dibutyl phthalate (DBP), BBP, DEHP, and di-n-octyl phthalate (DnOP) were also listed as priority environmental pollutants. Therefore, monitoring PAHs and PAEs in seafoods is necessary for food safety [20]. Among PAHs in fishes collected from Timsah Lake (Egypt), four carcinogenic compounds, including indeno(1,2,3-cd)pyrene, benzo(a)pyrene, dibenzo(a,h)anthracene, and benzo(b)fluoranthene, were detected. Another study in Brazil also showed that benzo[a]pyrene and dibenzo[a,h]anthracene was found in three species of shellfish, raising significant concerns about the safety of seafood consumption [21]. In Bohai Bay and Songhua River (China), the concentration of di(2-ethylhexyl) phthalate in shellfish and benthos showed high values that may pose a potential carcinogenic risk through consumption [22].

Studies on PAH and PAE contamination in seawater, marine sediment, and seafood from Viet Nam were limited. The previous study indicated that PAH concentrations in mollusk species commonly consumed as seafood were relatively low, ranging from 56 to 246 ng g^−1^ [23]. For the marine finfishes, phthalate concentrations in samples collected along coast of Viet Nam showed a mean value of 8.2 ng g^−1^ [24]. Therefore, the present study aims to provide an understanding of the PAH and PAE contamination in farmed species along the coast of Khanh Hoa, Viet Nam. The average daily intake (ADI), hazard quotient (HQ), hazard index (HI), and incremental lifetime cancer risk (ILCR) were calculated for the health risk assessment.

## 2. Materials and Methods

### 2.1. Study Sites

**Figure 1:**
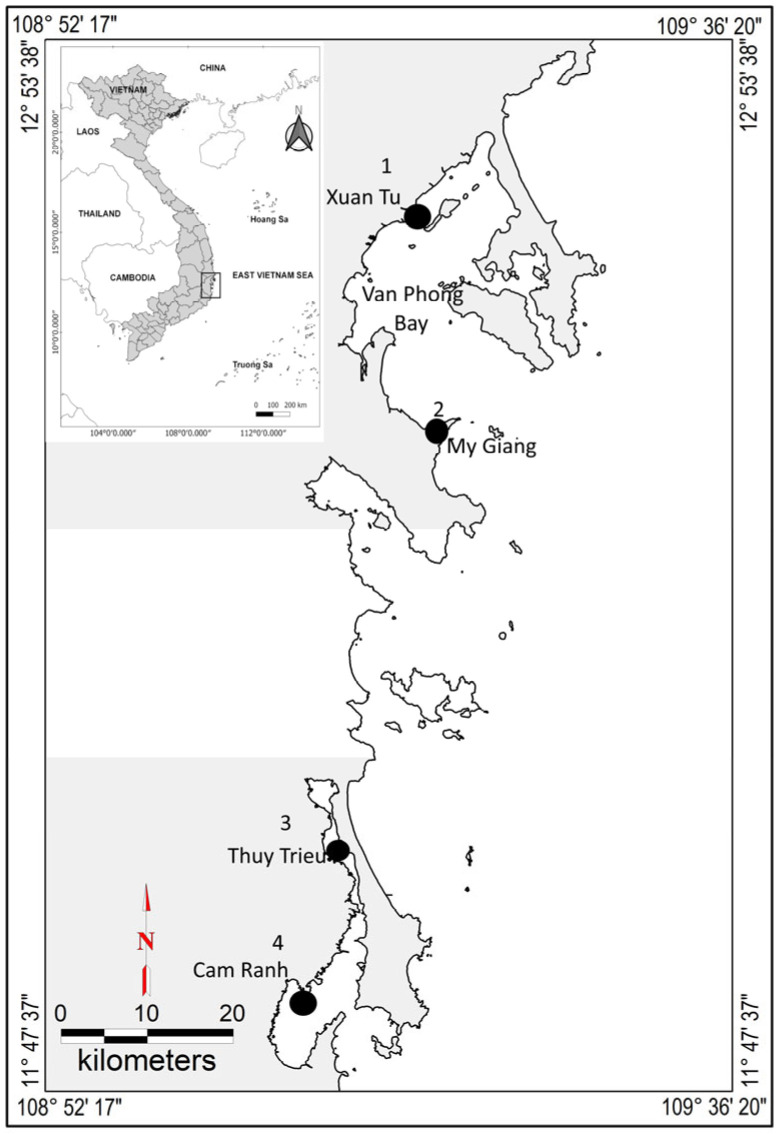
Map of the study area and location of the sampling sites. 1, Xuan Tu (XT); 2, My Giang (MG); 3, Thuy Trieu (TT); 4, Cam Ranh (CR).

Khanh Hoa province is located in central Viet Nam; four sampling sites, including Xuan Tu (XT), My Giang (MG), Thuy Trieu (TT), and Cam Ranh (CR), were selected due to several farming activities (Figure 1). The distances between these sampling sites are from 20 to 85 km. Among the four sampling sites, XT and MG are located in Van Phong Bay, in the northern region of the province. Van Phong Bay is a semi-enclosed bay expanding to approximately 150,000 hectares; the maximum depth is about 34 m. Located on the western side of the bay, XT serves as the main marine aquaculture activity in Khanh Hoa. MG is located on the southern side of the bay. In recent decades, the expansion of heavy industries—such as shipbuilding, thermal power generation, and aquaculture—has raised significant environmental concerns in the area. TT and CR are located in the southern part of Khanh Hoa. TT is a lagoon with limited water exchange that connects to Cam Ranh Bay. The lagoon has a maximum depth of about 6 m, and fish farms are distributed along its shoreline. CR is a semi-enclosed water body covering approximately 12,000 hectares. It connects with TT in the north and opens to the South China Sea in the east. This area supports various activities, including heavy industry, port operations, and marine aquaculture. Commonly farmed species in sampling sites were Pacific white shrimp (*Litopenaeus vannamei*), spotted babylon (*Babylonia areolata*), snubnose pompano (*Trachinotus blochii*), and giant grouper (*Epinephelus lanceolatus*). The cockle (*Marcia hiantina*) is also frequently found in the mudflats of TT. Pacific white shrimp were cultured in four locations, whereas spotted babylon and snubnose pompano were not found in TT. Therefore, other farmed fish (giant grouper) and mollusk (cockle) collected from TT were used as substitutes for spotted babylon and snubnose pompano.

### 2.2. Sampling and Extraction

Pacific white shrimp were collected from four sampling sites: XT, MG, TT, and CR. At each site, samples were obtained from three separate farms, with three individuals collected per farm. The average weight of harvest-sized shrimp was approximately 18 g. Fifteen individuals of snubnose pompano were sampled from fish cages at XT, MG, and CR, with an average weight of around 850 g. Similarly, fifteen spotted babylons, each weighing between 6 and 8 g, were collected from the same three sites. Giant grouper and cockle samples were collected exclusively from TT. Five giant groupers, each weighing between 1.2 and 1.5 kg, were sampled from a local fish farm, while five cockle specimens were gathered from mudflats, with wet weights ranging from 23 to 24 g. In total, 76 samples across five species were collected for analysis. All samples were stored in iceboxes and transported to the laboratory on the same day. All samples were collected during March and April 2023.

Only muscle tissues were used for analysis and were carefully separated using a stainless steel knife. An aliquot of 0.5 g of homogenized, freeze-dried tissue was extracted using water bath ultrasonication with 5 mL of an acetone/n-hexane mixture (1:1, v/v). To separate the solid and liquid phases, the extract was centrifuged at 2500× *g* for 10 min. The supernatant was collected and repeated to ensure maximum recovery. The combined extracts were desulfurized using activated copper and dehydrated with anhydrous sodium sulfate. The final extract was concentrated to 0.5 mL under a gentle stream of nitrogen, and 200 ng of a surrogate standard mixture was added prior to instrumental analysis.

### 2.3. Sample Analysis

The PAHs and PAEs were analyzed through a triple quad gas chromatograph–mass spectrometry GC/MS-TQ8040 (Shimadzu, Kyoto, Japan) equipped with a DB-5ms column. The temperature program began at 110 °C, with gradual increases to a final temperature of 310 °C. Helium was used as the carrier gas. The MS detector used electron impact (EI) mode with selective ion monitoring (SIM) for quantification. The injection volume was 1 μL. All analytical data were subjected to strict quality control. The target of 12 PAH compounds includes Acenaphthylene (Acy), Fluorene (Flu), Phenanthrene (Phe), Anthracene (Ant), Pyrene (Pyr), Chrysene (Chr), Benzo[a]anthracene (BaA), Benzo[b]fluoranthene (BbF), Benzo[k]fluoranthene (BkF), Indeno[1,2,3-cd] pyrene (IP), Dibenzo[a,h]anthracene (DA), and Benzo[g,h,i] perylene (BP). The target of 6 PAE compounds includes Dimethyl phthalate (DMP), Dibutyl phthalate (DBP), Diethyl Phthalate (DEP), butyl benzyl phthalate (BBP), Di(2-ethylhexyl) phthalate (DEHP), and Di(2-ethylhexyl) adipate (DEHA). The method blanks and spiked samples were analyzed along with each set of samples. The GC/MS-TQ8040 was calibrated using standards that were run with each batch of samples. The standard solutions were diluted to obtain five different PAH concentration levels: 1, 2, 5, 10, and 20 µg kg^−1^. Measurement was conducted immediately after the stock standard was prepared. These solutions were analyzed for each batch of 15 samples. In the blank solutions, PAH molecules were present at very low concentrations (<0.5 ng mL^−1^) or were not detectable. The recovery for the certified reference material (SRM 1974c, organics in muscle tissue of *Mytilus edulis*, GMA, Washington, DC, USA) was between 73.1 ± 4.7% and 104.8 ± 5.6%. The analysis results for the procedural blank were all lower than the detection limit. The precision was evaluated as the relative standard deviation (RSD) of the measured results. The calibration curve equations, RSD (%), limit of detection (LOD), and limit of quantification (LOQ) of the targeted 12 PAH and 6 PAEs are presented in Appendix A, Table A1.

### 2.4. Statistical Analysis

The results are presented as mean ± standard deviation. The variability among three groups (fish, mollusk, and shrimp) within locations, and within species (Pacific white shrimp, snubnose pompano, and spotted babylon) among four or three locations was assessed by performing one-way analysis of variance with a significance level at *p*-value < 0.05. The IBM SPSS Statistic version 23 was used for analyses.

### 2.5. Health Risk Assessment

The presence of PAEs in farmed species suggests that exposure occurs predominantly through the food consumption. Therefore, it is necessary to assess the potential health risks caused by PEAs to human health. In this study, the average daily intake (ADI) (Formula (1)), hazard quotient (HQ) (Formula (2)), and hazard index (HI) (Formula (3)) [25] were calculated as follows:

(1) $$ADI=\frac{IR\times C_{PEA}\times ED}{BW\times AT}$$

(2) $$HQ=\frac{ADI}{RfD}$$

(3) $$HI=\sum_{i=1}^{n}{HQ}_{i}$$

where IR is the seafood consumption rate for an individual (0.055 kg day^−1^, CPEA (mg kg^−1^) is the PEA concentration, ED (years) is exposure duration, which is quantified as 34.5 for adults, BW (kg) is the mean body weight of adults (60 kg), and AT is the average lifetime (70 years). RfD (mg kg^−1^ day^−1^) is reference dose. HI < 1, adverse effects are unlikely. HI ≥ 1, there is a potential health risk.

The incremental lifetime cancer risk (ILCR) is computed as the increased likelihood of an individual developing cancer over their lifetime due to exposure to a suspected carcinogen [26]. The ILCR values related to the dietary consumption of carcinogenic PAHs were calculated using the following equation:

(4) $$Cancer\;Risk=\;\frac{C\;\times \;IR\;\times \;EF\;\times \;ED\;\times \;CF\;}{BW\;\times \;{AT}_{ca}}$$

where CR is the cancer risk; EF represents the exposure frequency (24 days/year for the flexitarian; 365 days/year for the fish-eating population and entire population); ED stands for exposure duration, which is taken as 30 years for adults [27]; CF represents the conversion factor, which is 1 × 10^−6^ kg mg^−1^; OSF is the cancer oral slope factor of Acy, Flo, Phe, Pyr = 0.001; BaA, IP = 0.1. ATca is the average time for carcinogenic PAHs (70 years for adults). The cancer risk is considered negligible in the case of CR < 10^−6^, acceptable in the case of 10^−6^ < CR< 10^−4^, and unacceptable in the case of CR > 10^−4^ [28,29].

## 3. Results

### 3.1. Occurrence of the PAEs in Farmed Species

**Figure 2:**
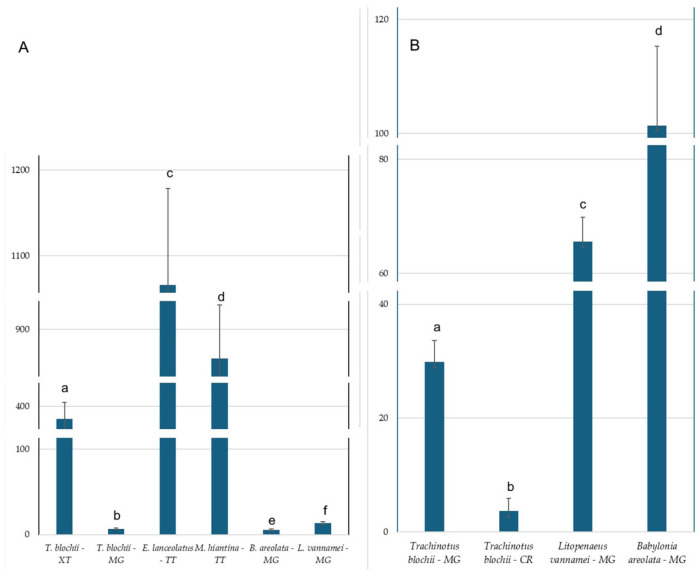
Concentration of DEHP (**A**) and DEP (**B**) in tissues from the farmed species along the coast of Khanh Hoa province. Different letters (a–f) printed within the same column show significantly different means of observed data (*p* < 0.05) according to post hoc Tukey’s HSD test. Data are presented in mean ± SD. See Figure 1 for abbreviation of locations.

The concentrations of phthalate esters (PAEs) in farmed species from four sampling sites exhibited considerable variation. In Khanh Hoa, the detection frequencies of six PAEs in farmed species followed the descending order: DEHP (55.6%), DEP (44.4%), BBP (33.5%), DMP and DBP (11.1% each). DEHA was not detected in any samples. The total concentration of six PAEs (Σ6PAEs) ranged from 7.76 µg kg^−1^ in spotted babylon to 1.066 µg kg^−1^ in giant grouper. In site 1 (XT), DBP (4.57 ± 0.65 µg kg^−1^) and DEHP (385 ± 19.70 µg kg^−1^) were detected in snubnose pompano, while DMP, and BBP were found in spotted babylon. PEAs were not found in Pacific shrimp. DEHP was the predominant compound, accounting for 97% of the total PAEs (Σ3PAEs) in this area. In spotted babylon, the concentrations of DMP and BBP were 3.06 ± 0.39 µg kg^−1^ and 4.09 ± 0.41 µg kg^−1^, respectively. In samples collected from MG, DEHP, DEP, and DBP were detected in all three farmed species. DEP was the dominant compound across all species, with concentrations of 29.83 ± 3.80 µg kg^−1^ (60.09% of total PAEs) in snubnose pompano, 65.55 ± 4.27 µg kg^−1^ (81.9%) in Pacific white shrimp, and 101.37 ± 13.97 µg kg^−1^ (92.7%) in spotted babylon. BBP was detected only in snubnose pompano, with a concentration of 12.96 ± 1.73 µg kg^−1^. DEHP concentrations were slightly higher in Pacific white shrimp (13.3 ± 1.62 µg kg^−1^) than in snubnose pompano (6.85 ± 0.61 µg kg^−1^) and spotted babylon (5.37 ± 1.24 µg kg^−1^). In farmed species collected from two sites in the southern part of the province, five PAEs, including DEP, DBP, BBP, and DEHP, were detected. At TT, DEHP was the dominant PAE, with concentrations of 1.066 ± 112.6 µg kg^−1^ (100%) in giant grouper and 866 ± 62.6 µg kg^−1^ (100%) in cockle. Other PEAs were not found in Pacific white shrimp. At CR, BBP was the only PAE detected in both Pacific white shrimp and spotted babylon, with concentrations of 26.68 ± 3.06. In snubnose pompano, DEP and DBP were present at concentrations of 3.66 ± 0.82 µg kg^−1^ and 11.26 ± 2.25 µg kg^−1^, respectively. There were no PAEs in the tissue of spotted babylon. The results of one-way ANOVA showed significant differences of DEHP and DEP concentrations between species (Figure 2).

### 3.2. Occurrence of the PAHs in Farmed Species

**Figure 3:**
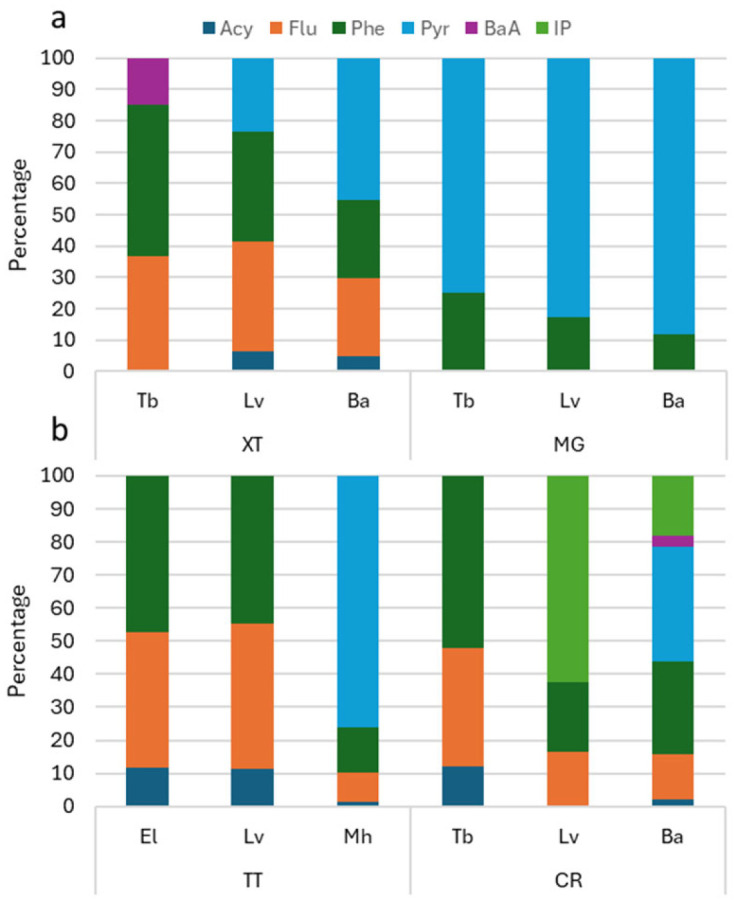
Average PAH composition in tissue of farmed species collected from coast of Khanh Hoa province: (**a**) two sites in northern part, (**b**) two sites in southern part. Acy: Acenaphthylene; Flu: Fluorene; Phe: Phenanthrene; Pyr: Pyrene; BaA: Benz[a]anthracene; IP: Indeno[1,2,3-cd] pyrene. See Figure 1 for abbreviation of locations.

The detection and diversity of polycyclic aromatic hydrocarbons (PAHs) varied significantly among species and sampling sites. Among the concentrations of 12 PAHs (Σ12PAHs) measured in the farmed species, 6 PAHs were detected, ranging from 9.14 µg kg^−1^ in Pacific white shrimp to 47.34 µg kg^−1^ in cockle. At the two northern sites (XT and MG), five PAHs, including acenaphthylene (Acy), fluorene (Flu), phenanthrene (Phe), pyrene (Pyr), and indeno[1,2,3-cd] pyrene (IP) were detected. In XT, Flu and Phe were present in nearly all samples from the three studied species (Table 1). It was observed that 3-ring PAHs (Phe and Flu) were the dominant contribution in all species with the mean percentage of 49.8% (in spotted babylon)–85.2% (in snubnose pompano), followed by 4-ring PAHs of Pyr (23.5–45.5%). The composition of 6-ring PAHs (IP) was low, accounting for 14% in snubnose pompano and zero in Pacific white shrimp and spotted babylon (Figure 3a). The average concentrations of Flu were 10.17, 3.33, and 3.42 µg kg^−1^ in snubnose pompano, Pacific white shrimp, and spotted babylon, respectively. Similarly, the concentrations of Phe in these species were 7.74, 3.36, and 3.48 µg kg^−1^, respectively. The average concentration of Pyr in spotted babylon (6.34 µg kg^−1^) was notably higher than in Pacific white shrimp (2.15 µg kg^−1^). Acy was also detected at low concentrations (<0.7 µg kg^−1^) in Pacific white shrimp and spotted babylon. Notably, indeno[1,2,3-cd] pyrene (IP), a potentially carcinogenic compound, was detected in snubnose pompano at a concentration of 3.1 µg kg^−1^. At site MG, two PAHs were found across all three species, with Pyr being the dominant compound. The highest concentration of Pyr was observed in spotted babylon (40.86 µg kg^−1^), followed by snubnose pompano (13.34 µg kg^−1^) and Pacific white shrimp (14.09 µg kg^−1^). The average concentrations of Phe were 5.47, 4.21, and 2.91 µg kg^−1^ in spotted babylon, snubnose pompano, and Pacific white shrimp, respectively (Table 1). Farmed fishes collected in MG showed a dominant contribution of 4-ring PAHs of Pyr with 75.1–88.2%, followed by 3-ring PAHs of Phe (11.8–24.9%) (Figure 3b).

At the two southern sites in the province (TT and CR), six out of twelve PAHs were detected across five farmed species. In TT, pyrene (Pyr) exhibited the highest average concentration, measured at 36.09 µg kg^−1^, and was detected only in cockle. Phenanthrene (Phe) concentrations were highest in giant grouper (7.79 µg kg^−1^), followed by cockle (6.36 µg kg^−1^) and Pacific white shrimp (4.09 µg kg^−1^). Similarly, fluorene (Flu) concentrations in giant grouper (6.70 µg kg^−1^) were higher than in the other two species, which ranged from 4.02 to 4.19 µg kg^−1^. Low concentrations of acenaphthylene (Acy), ranging from 0.69 to 1.95 µg kg^−1^, were also detected in these three species (Table 1). The composition patterns of PAHs in samples collected in TT revealed that 3-ring PAHs (Phe and Flu) are dominant with around 88% in Pacific white shrimp and giant grouper, followed by another 3-ring PAH (Acy, around 11%). However, 4-ring PAH (Pyr) was the dominant composition with 72.6%, followed by 3-ring PAHs: Phe (13.5%), Flu (8.9%), and Acy (1.5%) (Figure 2B). At site CR, spotted babylon exhibited both the highest PAH diversity and the highest overall concentrations. In contrast, snubnose pompano and Pacific white shrimp contained only three PAHs each. Specifically, in snubnose pompano, the average concentrations of Acy, Flu, and Phe were 3.06, 8.95, and 13.16 µg kg^−1^, respectively. In comparison, the concentrations of these compounds in spotted babylon were lower: 0.71 µg kg^−1^ (Acy), 4.55 µg kg^−1^ (Flu), and 9.22 µg kg^−1^ (Phe). In Pacific white shrimp, Acy was not detected, while Flu and Phe were present at average concentrations of 4.60 and 5.38 µg kg^−1^, respectively. Pyr and benzo[a]anthracene (BaA) were detected exclusively in spotted babylon, with concentrations of 11.56 and 1.10 µg kg^−1^, respectively. Indeno[1,2,3-cd] pyrene (IP) was found at a higher concentration in Pacific white shrimp (17.51 µg kg^−1^) and spotted babylon (5.95 µg kg^−1^) compared to snubnose pompano from site XT (Table 1).

For Acy, the results of one-way ANOVA showed significant differences in Acy between giant grouper and snubnose pompano collected from CR. However, there were no significant differences of Acy between Pacific white shrimp collected in XT and TT. For Flu, a significant difference was found between snubnose pompano collected in CR and giant grouper, but no significant difference was found between snubnose pompano collected in CR and XT. Within Pacific white shrimp samples, the Flu concentration showed a significant difference only between samples collected in XT and CR. The results of one-way ANOVA also showed no significant differences in Flu between cockle and spotted babylon collected in CR and MG. For Phe, a significant difference was found between giant grouper and snubnose pompano collected from three locations. Significant differences were also observed in Pacific white shrimp samples collected from four locations. The results of the one-way ANOVA revealed significant differences in Phe levels between cockle and spotted babylon collected in CR and XT, but no significant difference between cockle and spotted babylon collected in MG. For Pyr, a significant difference was found between Pacific white shrimp collected in XT and MG. Within the mollusk group, significant differences were observed among spotted babylon collected from three sites, and between cockle and spotted babylon (Table 1).

### 3.3. Health Risk Assessment

The average daily intake (ADI) of the sum of three phthalate esters (∑3PEAs: DBP, DEP, and DEHP) for adults was estimated at 2.2 × 10^−4^, 6.0 × 10^−4^ and 3.3 × 10^−2^ mg kg^−1^ day^−1^, respectively. The corresponding hazard quotient (HQ) values were 3.0 × 10^−7^ for DBP, 1.9 × 10^−7^ for DEP, and 1.6 × 10^−5^ for DEHP. The hazard index (HI) calculated in this study was 1.7 × 10^−5^, which is well below the threshold of 1, indicating no significant health risk. Among the different groups of farmed species: fishes (snubnose pompano and giant grouper), shrimp, and mollusks (spotted babylon and cockle), the highest ADI of DEHP was observed in mollusks (7.2 × 10^−2^ mg kg^−1^ day^−1^), followed by fishes (6.4 × 10^−2^ mg kg^−1^ day^−1^) and shrimp (2.2 × 10^−3^ mg kg^−1^ day^−1^). All HQ values for the three PEAs across these groups were below 1, and the calculated HI values also remained under 1 (Appendix A, Table A2), suggesting minimal risk. Slight variations in HQ values for non-carcinogenic effects of PAEs were noted across the four sampling locations. The highest HQ value for DEHP was recorded at TT (8.0 × 10^−5^), while values in the other locations ranged from 5.7 × 10^−7^ to 3.3 × 10^−5^. The total HI, calculated as the sum of HQ values for the three PEAs, remained significantly below 1 across all locations. Detailed ADI, HQ, and HI values for adults from the four sampling sites are presented in Appendix A, Table A3.

**Figure 4:**
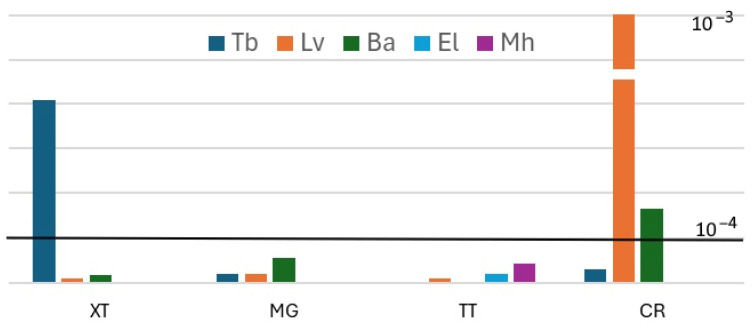
Estimated ILCRs from consuming the farmed species collected from four sites in Khanh Hoa province. Dark line: Maximum acceptable risk level. See Table 1, Table 2 for abbreviation of scientific name; Figure 1 for abbreviation of locations.

The Incremental Lifetime Cancer Risk (ILCR) has been widely used to evaluate the risk of PAHs. The result showed that ILCR values of farmed species collected from MG and TT were acceptable in this case (1.1–5.4 × 10^−5^). However, ILCR values from spotted babylon collected in CR (1.6 × 10^−4^) and snubnose pompano collected in XT (4.1 × 10^−4^) were slightly higher than maximum acceptable risk level. Specially, samples of Pacific white shrimp collected in CR showed the highest values of ILCR (1.6 × 10^−3^) or an unacceptable level (Figure 4).

## 4. Discussion

In this study, the five farmed species collected from four sites along the coast of Khanh Hoa, Viet Nam, were found to contain five of the six examined PAEs. The total concentration of PAEs in this study was matched by those of the fish from Bohai Bay [22], Hangzhou Bay [30], and Hainan coast, China [31], but higher than those reported in natural marine fish collected in Viet Nam (mean concentration of 8.2 ng g^−1^) [24]. In this study, giant grouper—a demersal fish—and cockle showed higher PAEs, whereas snubnose pompano revealed high variation in PAEs in different sites. In TT, farmers often use trash fish to feed giant grouper; the PAEs present in the trash fish may accumulate in the giant grouper. Therefore, it could explain the higher PAE concentration in giant grouper. According to Savoca et al. [32], the habitat, along with the feeding habits and preferences of the species, are key factors influencing the uptake of phthalates from the marine environment and their accumulation in various tissues. Study of Marmara et al. [33] in the Ionian Sea, Italy, showed that the demersal species such as *Mullus barbatus* tend to accumulate higher levels of hydrophobic phthalates. The higher PEAs in cockle found in this study may be explained by the fact that cockle is a filter-feeding organism. It is a species that was proposed as a global bioindicator of microplastic pollution [34,35]. In this study, both species, including giant grouper and cockle collected in TT, showed higher PAE concentration, which may reflect higher microplastic and PAEs in the environment. Therefore, due to microplastic and PAEs in the environment, natural fishes in TT should be studied for better understanding.

In this study, DEHP was found to be the most dominant pollutant and exhibited the highest average concentration. Several previous studies on the occurrence of PAEs in various fish species also reported similar findings. For example, DEHP concentrations in fish tissues collected in the northern Aegean Sea in Greece and the western Ionian Sea in Italy ranged from 13.7 to 19.4 ng g^−1^, much higher than other PEAs [33]. Recently, Liu et al. [22] also showed that DEHP (2.152 ng g^−1^) was a predominant chemical of PAEs in marine fishes collected from Bohai Bay, China. DEHP was the most abundant compound found in both freshwater and marine fish in Taiwan, and the marine finfish (yellow croaker) showed highest DEHP in tissue (4.26 µg g^−1^) [36]. Recent evidence indicates that microplastics act as effective vectors, adsorbing PEAs from surrounding waters and transporting them across ecosystem [8]. Therefore, phthalate esters have been considered indicators of microplastic contamination [37]. Cao et al. [38] indicated that microplastics is a major source of phthalate esters in aquatic environments.

The results from this study also indicated that the PAH concentrations (13.92–47.34 µg kg^−1^) in two species of mollusk (spotted babylon and cockle) were generally higher than those in fishes (snubnose pompano and giant grouper) and crustacean (Pacific white shrimp. In comparison with the previous study, Hoang et al. [39] reported that the total PAH concentration in green mussel (*Perna viridis*) collected in Can Gio, Viet Nam, was lower, ranging from 3.65 to 15.79 µg kg^−1^. Another study on PAH concentration in oysters showed that it can reach up to 64.45 µg kg^−1^. However, gastropods showed a lower concentration than our results [40]. According to Silva et al. [41], the PAH concentrations in commercial marine bivalves revealed significant variation among species. For example, PAH concentrations in *Crassostrea belcheri* collected from Malaysia were from 309 to 2225 µg kg^−1^, whereas *Meretrix lusoria* collected in Taiwan showed lower concentrations (10.18 to 31.49 µg kg^−1^) [29]. Another marine bivalve species—*Callista chione*—collected in Morocco showed from 1 to 51 µg kg^−1^ [42]. For finfish, the PAH concentrations in this study were lower than those reported in freshwater fishes (22–228 µg kg^−1^) collected in Ha Noi, Viet Nam [43]. Baumard et al. [44] indicated that total PAH concentration lower than 100 ng g^−1^ is considered a low contamination level. Therefore, the farmed species analyzed in this study were weakly contaminated by PAHs compared to other aquatic organisms worldwide. A comparison of PAH concentrations in the farmed species from this study and previous works is presented in Table 2. Note that this comparison is relative due to differences in extraction, detection, and quantification methods. Most previous studies used lyophilization or freeze-drying of tissues for extraction, which appears to be a common pre-treatment step to remove water from samples. This procedure helps preserve the compounds present and increases the efficiency of PAH extraction. A wide variety of organic solvents have been used for PAH and PEA extraction, such as an acetone/dichloromethane mixture [45], acetone/n-hexane [29], cyclohexane [46], acetone [47], HCl/Chloroform [39], mixture of acetone, dichloromethane, and n-hexane [48]. The clean-up step is essential to remove suspended solids and other potential interferences. Column chromatography, organic solvent dispersive solid-phase extraction, and gel permeation chromatography were commonly used. In this study, the detection of PAHs and PEAs in marine organisms was performed using a high-sensitive analytical technique, gas chromatography–tandem mass spectrometry (GC-MS/MS). Other techniques, such as gas chromatography–mass spectrometry (GC–MS), gas chromatography with flame ionization detector (GC-FID), and high-performance liquid chromatography with fluorescence detector (HPLC-FLD), have also been widely applied in previous works.

**Table 2:** Comparison of PAH concentrations (µg kg^−1^) in the marine organisms collected in Khanh Hoa and other locations. 1 = Sample: muscle tissues. Extraction: water bath ultrasonication using an acetone:n-hexane mixture (1:1, v/v). Detection and Quantification: GC–MS/MS, and 2 = Sample: muscle tissues. Extraction: Soxhlet extraction using an acetone and dichloromethane mixture (1:1, v/v). Detection and Quantification: GC–MS.

Species/Name	Location	∑PAHs	Sample Processing	Sources
Fishes				
***Trachinotus blochii***	**Viet Nam**	**17.6–25.7**	Sample: muscle tissues Extraction: water bath ultrasonication using an acetone/n-hexane mixture (1:1, v/v) Detection and Quantification: GC–MS/MS ^1^ 1	**This study**
*Trachinotus blochii*	Hong Kong	48.7–67.2	Sample: muscle tissues Extraction: Soxhlet extraction using an acetone and dichloromethane mixture (1:1, v/v) Detection and Quantification: GC–MS ^2^	[45] Cheung
***Epinephelus lanceolatus***	**Viet Nam**	**16.43**	**^1^**	**This study**
*Epinephelus coioides*	Hong Kong	30.2–50.2	^2^	[45]
*Epinephelus bleekeri*	Hong Kong	37.6–45.8	^2^	[45]
*Lateolabrax japonicus*	Taiwan	35.16	Sample: muscle tissues Extraction: water bath ultrasonication with the mixture of acetone/n-hexane (1:1, v/v) Detection and Quantification: GC–MS	[29]
Freshwater fishes	Viet Nam	22–228	Sample: muscle tissues Extraction: ultrasound with the n-hexane and dichloromethane mix solvent. Detection and Quantification: GC × GC-TOF/MS	[43]
Mollusks				
***Babylonia areolata***	**Viet Nam**	**13.32–46.33**	**^1^**	**This study**
***Marcia hiantina***	**Viet Nam**	**47.34**	**^1^**	**This study**
*Crassostrea belcheri*	Malaysia	309–2225	Sample: soft tissues Extraction: cyclohexane Detection and Quantification: GC-MS	[46]
*Crassostrea gigas*	Japan	289–450	Sample: soft tissues Extraction: acetone Detection and Quantification: GC-MS	[47]
*Perna viridis*	Viet Nam	3.65–15.79	Sample: soft tissues Extraction: HCl/Chloroform Detection and Quantification: HPLC-FLD	[39]
*Perna viridis*	Viet Nam	34–110	Sample: soft tissues Extraction: mixture of acetone, dichloromethane, n-hexane (1:1:1, v/v/v) in a Soxhlet apparatus Detection and Quantification: GC-MS	[48]
Crustaceans				
***Litopenaeus vannamei***	**Viet Nam**	**9.14–27.94**	**^1^**	**This study**
*Metapenaeus affinis*	Iran	1644–3792	Extraction: Soxhlet system using dichloromethane Detection and Quantification: GC-MS	[49]
*Portunus trituberculatus*	China	119.11	Sample: edible tissues Extraction: Soxhlet system using n-hexane/acetone (1:1 v/v) Detection and Quantification: GC-MS/MS	[50]

Regarding PAH composition profiles, this study was similar among the farmed species with 3-ring and 4-ring PAHs. The findings from this study are also consistent with previous studies in the world. For example, shrimp collected in the northwest Persian Gulf were found to contain a higher percentage of low-molecular-weight PAHs (2- and 3-ring) than high-molecular-weight PAHs (5- and 6-ring) [49]. The low-molecular-weight PAHs were also reported in marine organisms collected in Zhejiang, Tanmen, and Zhuhai (China) [3,51]. Zhang et al. [50] indicated that 2- and 3-ring PAHs were identified as the main congeners in marine organisms collected from two fishing grounds in China. This study revealed that spotted babylon and cockle accumulated higher levels of PAHs compared to fish and shrimp. This is likely due to their life cycle contact with bottom sediments. Several studies have demonstrated that marine sediments serve as the primary sinks for PAHs, and PAH in mollusk is often higher than other marine animals [52,53].

The BaA congener was detected in only one sample of spotted babylon collected in CR, with a low concentration. In contrast, IP was found in three species: snubnose pompano collected in XT, spotted babylon, and Pacific white shrimp collected in MG. The International Agency for Research on Cancer [54] suggested that BaA and IP are two of six congeners (group 2B). A member of the group is classified as possibly carcinogenic to humans. These congeners are known as good indicators of the carcinogenic potency of PAHs in food [55]. In Viet Nam, the previous study on green mussels showed that BaA was not detected [39]. In Taiwan, Ju et al. [29] revealed that BaA presented at low concentrations in freshwater clams (*Corbicula fluminea formosa*), but was not detected in three finfish species or other clams. Another study in China also indicated that BaA was not found in six finfish species and two crustacean species collected from the coastal waters of Zhejiang [3]. In contrast, marine finfish such as Belanger’s croaker collected from the East China Sea contained high BaA concentrations with 24.13 µg kg^−1^ [56]. For IP, compared with the previous studies, the concentration of IP congener found in Pacific white shrimp tissue collected in CR (17.51 µg kg^−1^) from this study was considerably higher than the levels reported in crustaceans from China (0.11 µg kg^−1^) [3], and octopus collected in northeast Brazil (5.35 µg kg^−1^) [57]. European Union regulation (EC) No. 1881/2006 set the allowable maximum levels for PAHs in foodstuffs at 2.0 ng g^−1^ for BaP in fish muscle and 5.0 ng g^−1^ in bivalve mollusks, as well as 12.0 ng g^−1^ for the sum of four PAH congeners (BaA, CH, BaP, and BbF) in fish muscle and 30.0 ng g^−1^ in bivalve mollusks [58]. In this study, BaP was not detected in any samples. The sum of four PAH congeners (BaA, CH, BaP, and BbF) in spotted babylon was 1.10 µg kg^−1^, which is much lower than the allowable levels.

The ILCR values of PAHs for the consumption of farmed species in this study ranged from 1.1 × 10^−5^ to 2.2 × 10^−3^. In the previous studies, the ILCR value showed a variation among species and location/country. For example, the ILCR values of PAHs for fish consumption in Taiwan were 3.87 × 10^− 7^ to 6.13 × 10^−4^ [29] or 1.51 × 10^−6^ to 1.20 × 10^−5^ in China [48]. However, Said et al. [59] reported that the risk level from fish consumption in Saudi Arabia was 2.1 × 10^−3^–1.25 × 10^−2^. The lifetime excess cancer risk via fish consumption in Haimen bay (China) was higher than the serious risk level [60]. It is well known that the risk management decisions are most frequently based on the cancer risk range of 10^−6^–10^−4^ [61]. Two of the three species in CR and one species in XT showed ILCR values higher than 10^−4^. The ILCR values of PAHs in this study were calculated based on the fresh samples. Therefore, the results indicate that the long-term consumption of these fish species may pose a cancer risk to consumers. However, cooking and digestion processes can significantly reduce the bioavailability of PAH and PAE. For example, steam precooking has been shown to reduce PAH contamination in traditionally smoked shrimp [62]. In contrast, the charcoal-grilled method can increase PAHs in the dishes [63]. PAHs in farmed fish mainly originate from environmental contamination. Thus, several environmental control strategies should be implemented in XT and CR, including reducing petroleum pollution, industrial runoff, and urban wastewater entering cultivation areas. In addition, aquaculture water and marine sediment should be regularly monitored, and farming should be avoided in heavily contaminated areas. To obtain a clear understanding of PAH and PEA contamination along Khanh Hoa province, natural fish, environmental samples (seawater and marine sediment), and other information on natural conditions will be collected for analyses.

## 5. Conclusions

This study presents the most up-to-date findings on pollution levels and health risk assessments of six PAEs and twelve PAHs in five farmed fish species collected from four coastal sites in Khanh Hoa province, Viet Nam. Among the PAEs, DEHP exhibited the highest mean concentration and was detected in most samples from aquaculture sites, while DEP and BBP showed higher concentrations in samples collected from industrial areas. The hazard index (HI) calculated in this study was significantly below the safety threshold of 1, indicating no significant health risk. The ΣPAH concentrations were classified as low contamination levels. However, frequent consumption of snubnose pompano from XT, as well as spotted babylon and Pacific white shrimp from CR, may pose health risks, as the ILCR values for PAHs in these species exceeded the acceptable threshold. Future studies should investigate the presence of PAEs and PAHs in natural marine organisms and the surrounding marine environment, including sediments and seawater. This is the first study to report on the levels and health risks of PAEs and PAHs in farmed fish along the coast of Khanh Hoa province, underscoring the need for continued research on seafood safety in this region.

## Figures and Tables

**Table 1:** PAH concentrations (µg kg^−1^) in farmed species collected from XT and MG. ND = means of measured level below the method detection limit. *: detected in one sample. *Tb* = *Trachinotus blochii* (snubnose pompano), *Lv* = *Litopenaeus vannamei* (Pacific white shrimp), *Ba* = *Babylonia areolata* (spotted babylon). *El = Epinephelus lanceolatus* (giant grouper), *MH = Marcia hiantina* (cockle). Different letters (a–d) printed within the same column show significantly different means of observed data (*p* < 0.05) according to post hoc Tukey’s HSD test. Data are presented in mean ± SD. See Figure 1 for abbreviation of locations.

PAHs	Abb.	Fish			
		*Tb*-XT	*Tb*-MG	*Tb*-CR	*El*-TT
Acenaphthylene	Acy	ND	ND	3.06 ± 0.13 ^b^	1.95 ± 0.10 ^a^
Fluorene	Flu	7.74 ± 1.58 ^a^	ND	8.95 ± 0.25 ^ab^	6.70 ± 0.22 ^ab^
Phenanthrene	Phe	10.17 ± 1.86 ^a^	4.21 ± 0.64 ^b^	13.16 ± 050 ^d^	7.78 ± 0.40 ^c^
Pyrene	Pyr	ND	13.34 ± 0.61	ND	ND
Benz[a]anthracene	BaA			ND	ND
Indeno[1,2,3-cd] pyrene	IP	3.10 ± 1.06	ND	ND	ND
PAHs	Abb.	Shrimp			
		*Lv-XT*	*Lv-MG*	*Lv-TT*	*Lv-CR*
Acenaphthylene	Acy	0.62 ± 0.29 ^a^	ND	1.03 ± 0.13 ^a^	ND
Fluorene	Flu	3.36 ± 0.65 ^a^	ND	4.02 ± 0.42 ^b^	4.60 ± 0.55 ^a^
Phenanthrene	Phe	3.33 ± 0.92 ^a^	2.91 ± 0.19 ^a^	4.09 ± 0.20 ^ab^	5.83 ± 0.37 ^abc^
Pyrene	Pyr	2.25 ± 0.36 ^a^	14.09 ± 0.35 ^b^	ND	ND
Benz[a]anthracene	BaA	ND	ND	ND	ND
Indeno[1,2,3-cd] pyrene	IP	ND	ND	ND	17.51 ± 2.05
PAHs	Abb.	Mollusk			
		*Ba*-XT	*Ba*-MG	*Ba*-CR	*Mh*-TT
Acenaphthylene	Acy	0.68 ± 0.10 ^a^	ND	0,71*	0.69 ± 0.06 ^a^
Fluorene	Flu	3.48 ± 0.72 ^a^	ND	4.55 ± 0.67 ^a^	4.19 ± 0.29 ^a^
Phenanthrene	Phe	3.42 ±0.97 ^a^	5.47 ± 0.58 ^ab^	9.22 ± 1.74 ^abc^	6.37 ± 0.56 ^ab^
Pyrene	Pyr	6.34 ±2.15 ^a^	40.86 ± 2.90 ^b^	11.56 ± 1.47 ^c^	36.09 ± 1.24 ^d^
Benz[a]anthracene	BaA	ND		1.10 *	ND
Indeno[1,2,3-cd] pyrene	IP	ND		5.95 ± 2.45	ND

## Data Availability

The original contributions presented in this study are included in the article. Further inquiries can be directed to the corresponding author.

## Abbreviations

The following abbreviations are used in this manuscript:

Acy	Acenaphthylene
ADI	Average Daily Intake
Ant	Anthracene
Ba	*Babylonia areolata*
BaA	benzo[a]anthracene
BBP	butyl benzyl phthalate
BbF	benzo[b]fluoranthene
BkF	benzo[k]fluoranthene
BP	benzo[g,h,i] perylene
Chr	chrysene
CR	Cam Ranh
DA	dibenzo[a,h]anthracene
DBP	dibutyl phthalate
DEHA	di(2-ethylhexyl) adipate
DEHP	bis(2-ethylhexyl) phthalate
DEP	diethyl phthalate
DA	dibenz[a,h]anthracene
DMP	dimethyl phthalate
DnOP	di-n-octyl phthalate
El	*Epinephelus lanceolatus*
Flu	Fluorene
GC-MS/MS	Gas Chromatograph–Mass Spectrometer
HI	Hazard Index
HQ	Hazard Quotient
IARC	International Agency for Research on Cancer
ILCR	Incremental Lifetime Cancer Risk
IP	Indeno[1,2,3-cd] pyrene
Lv	*Litopenaeus vannamei*
MDL	Method Detection Limit
Mh	*Marcia hiantina*
MG	My Giang
PAE	Phthalate Esters
PAH	Polycyclic Aromatic Hydrocarbons
Phe	Phenanthrene
Pyr	Pyrene
Tb	*Trachinotus blochii*
TT	Thuy Trieu
XT	Xuan Tu

## Appendix A

**Table A1:** LOD, LOQ, recovery, RSDs, and calibration equation for 12 PAHs and 6 PEAs.

PAHs	LOD (µg kg^−1^)	LOQ (µg kg^−1^)	Recovery (%)	RSD (%)	Calibration Equation
Acy	0.12	0.37	109.43	0.6–4.9	y = 1.0069x + 0.1188
Flu	0.08	0.28	86.32	1.5–5.2	y = 1.1624x − 0.3277
Phe	0.12	0.40	92.44	0.8–5.5	y = 1.0952x − 0.2113
Ant	0.09	0.31	98.94	1.4–6.4	y = 0.9673x + 0.0846
Pyr	0.17	0.54	90.99	0.8–6.2	y = 0.9683x + 0.135
Chr	0.21	0.65	94.72	2.3–6.5	y = 0.9743x + 0.0594
BaA	0.20	0.61	93.98	1.8–5.6	y = 1.0555x − 0.101
BbF	0.15	0.42	95.95	2.0–6.4	y = 1.084x − 0.1535
BkF	0.14	0.51	91.17	1.5–5.7	y = 1.0531x − 0.1488
IP	0.14	0.45	95.36	0.4–5.5	y = 1.0336x − 0.0323
DA	0.17	0.57	96.75	1.9–6.3	y = 0.9507x + 0.0325
BP	0.31	0.89	91.28	0.7–5.7	y = 0.9391x + 0.3194
PEAs					
DMP	0.75	2.48	109.62	3.5–9.6	y = 1.1541x − 0.2843
DBP	0.62	2.07	98.45	2.5–10.8	y = 0.9607x + 0.2362
DEP	0.71	2.33	103.76	3.0–11.7	y = 0.9641x + 0.1654
BBP	0.98	3.23	95.31	2.8–11.5	y = 1.0178x − 0.1243
DEHP	0.12	0.41	111.84	5.7–14.8	y = 1.1781x − 0.4312

**Table A2:** The average daily intake (ADI), hazard quotient (HQ), and hazard index (HI) were calculated for different types of farmed fish based on their consumption from coastal areas of Khanh Hoa.

		*C_PEA_*		*ADI*		*HQ*	
Overall	R*f*D (mg kg^−1^ day^−1^)	95th	Mean	95th	Mean	95th	Mean
DBP	752	9.6 × 10^−3^	1.4 × 10^−3^	1.6 × 10^−3^	2.3 × 10^−3^	2.1 × 10^−6^	3.0 × 10^−7^
DEP	3160	9.1 × 10^−2^	3.7 × 10^−3^	1.5 × 10^−2^	6 × 10^−3^	4.7 × 10^−6^	1.9 × 10^−7^
DEHP	2000	1.0	0.2	0.17	0.03	8.4 × 10^−5^	1.6 × 10^−5^
*HI*						9.1 × 10^−5^	1.7 × 10^−5^
Fishes							
DBP	752	1.0 × 10^−2^	4.6 × 10^−3^	1.7 × 10^−3^	7.5 × 10^−4^	2.3 × 10^−6^	1.0 × 10^−6^
DEP	3160	2.9 × 10^−2^	1.7 × 10^−2^	4.7 × 10^−3^	2.8 × 10^−3^	1.5 × 10^−6^	8.7 × 10^−7^
DEHP	2000	1.0	0.39	0.16	6.4 × 10^−2^	8.2 × 10^−5^	3.2 × 10^−5^
*HI*						8.6 × 10^−5^	3.4 × 10^−5^
Crustacean							
DBP	752	1.3 × 10^−3^	1.3 × 10^−3^	2.1 × 10^−4^	2.1 × 10^−4^	2.8 × 10^−7^	2.7 × 10^−7^
DEP	3160	6.2 × 10^−2^	3.3 × 10^−2^	1.0 × 10^−2^	5.5 × 10^−3^	3.3 × 10^−6^	1.7 × 10^−6^
DEHP	2000	1.3 × 10^−2^	1.3 × 10^−2^	2.2 × 10^−3^	2.2 × 10^−3^	1.0 × 10^−6^	1.1 × 10^−6^
*HI*						4.6 × 10^−6^	3.1 × 10^−6^
Mollusk							
DBP	752	1.3 × 10^−3^	1.3 × 10^−3^	2.2 × 10^−4^	2.2 × 10^−4^	2.9 × 10^−7^	2.9 × 10^−7^
DEP	3160	9.1 × 10^−2^	1.4 × 10^−3^	1.5 × 10^−2^	2.3 × 10^−4^	4.8 × 10^−6^	7.1 × 10^−8^
DEHP	2000	0.82	0.44	0.14	7.2 × 10^−2^	6.8 × 10^−5^	3.6 × 10^−5^
*HI*						7.3 × 10^−5^	3.6 × 10^−5^

**Table A3:** The average daily intake (ADI), hazard quotient (HQ), and hazard index (HI) were calculated for each location based on their consumption from coastal areas of Khanh Hoa.

		*C_PEA_*		*ADI*		*HQ*	
Xuan Tu	R*f*D (mg kg^−1^ day^−1^)	95th	Mean	95th	Mean	95th	Mean
DBP	752	4.4 × 10^−3^	4.6 × 10^−3^	7.5 × 10^−4^	7.5 × 10^−4^	1.0 × 10^−6^	1.0 × 10^−6^
DEP	3160	1.3 × 10^−3^	1.3 × 10^−3^	2.2 × 10^−4^	2.2 × 10^−4^	7.0 × 10^−8^	7.0 × 10^−8^
DEHP	2000	3.9 × 10^−1^	3.9 × 10^−1^	6.4 × 10^−3^	6.4 × 10^−3^	3.2 × 10^−5^	3.2 × 10^−5^
*HI*						3.3 × 10^−5^	3.3 × 10^−5^
My Giang							
DBP	752	1.4 × 10^−3^	1.3 × 10^−3^	2.3 × 10^−4^	2.2 × 10^−4^	3.1 × 10^−7^	2.9 × 10^−7^
DEP	3160	9.8 × 10^−2^	6.6 × 10^−2^	1.6 × 10^−2^	1.1 × 10^−2^	5.1 × 10^−6^	3.4 × 10^−6^
DEHP	2000	1.3 × 10^−2^	6.9 × 10^−3^	2.1 × 10^−3^	1.1 × 10^−3^	1.0 × 10^−6^	5.7 × 10^−7^
*HI*						6.5 × 10^−6^	4.3 × 10^−6^
Thuy Trieu							
DBP	752	1.3 × 10^−3^	1.3 × 10^−3^	2.1 × 10^−4^	2.1 × 10^−4^	2.8 × 10^−7^	2.9 × 10^−7^
DEP	3160	1.4 × 10^−2^	1.3 × 10^−3^	2.2 × 10^−4^	2.2 × 10^−3^	3.3 × 10^−6^	6.8 × 10^−8^
DEHP	2000	1.1	9.7 × 10^−1^	1.7 × 10^−1^	1.6 × 10^−1^	1.0 × 10^−6^	8.0 × 10^−5^
*HI*						4.6 × 10^−6^	8.0 × 10^−5^
Cam Ranh							
DBP	752	1.1 × 10^−2^	1.1 × 10^−2^	1.5 × 10^−3^	1.9 × 10^−3^	2.5 × 10^−6^	2.5 × 10^−6^
DEP	3160	3.7 × 10^−3^	3.7 × 10^−3^	4.9 × 10^−4^	6.0 × 10^−4^	1.9 × 10^−7^	1.9 × 10^−7^
DEHP	2000	0.0	0	0	0	0	0
*HI*						2.7 × 10^−6^	2.7 × 10^−6^
